# Deconvolution of synthetic mRNA expression: Nucleoside chemistry alters translatability

**DOI:** 10.1002/btm2.10622

**Published:** 2023-11-28

**Authors:** Hanieh Moradian, Marko Schwestka, Toralf Roch, Manfred Gossen

**Affiliations:** ^1^ Institute of Active Polymers, Helmholtz‐Zentrum Hereon Teltow Germany; ^2^ Berlin Institute of Health Center for Regenerative Therapies (BCRT) Berlin Germany; ^3^ CheckImmune GmbH, Campus Virchow Klinikum Berlin Germany

**Keywords:** in vitro transcribed messenger RNA (IVT‐mRNA), nucleic acid delivery, nucleoside chemistry, transgene expression, translation capacity

## Abstract

Recent technological advances in the production of in vitro transcribed messenger RNA (IVT‐mRNA) facilitate its clinical use as well as its application in basic research. In this regard, numerous chemical modifications, which are not naturally observed in endogenous mRNA, have been implemented primarily to address the issue of immunogenicity and improve its biological performance. However, recent findings suggested pronounced differences between expression levels of IVT‐mRNAs with different nucleoside modifications in transfected cells. Given the multistep process of IVT‐mRNA delivery and subsequent intracellular expression, it is unclear which step is influenced by IVT‐mRNA chemistry. Here, we deconvolute this process and show that the nucleoside modification does not interfere with complexation of carriers, their physicochemical properties, and extracellular stability, as exemplified by selected modifications. The immediate effect of mRNA chemistry on the efficiency of ribosomal protein synthesis as a contributor to differences in expression was quantified by in vitro cell‐free translation. Our results demonstrate that for the nucleoside modifications tested, translatability was the decisive step in determining overall protein production. Also of special importance for future work on rational selection of tailored synthetic mRNA chemistries, our findings set a workflow to identify potentially limiting, modification‐dependent steps in the complex delivery process.

**Translational Impact Statement**

The use of different mRNA chemistries, from established modifications to future chemical entities, might impact expression anywhere between transcript complexation and ribosomal translation. Thus, the rational optimization of nucleoside chemistry according to the intended application mandates that the complex process of introducing synthetic mRNAs into cells is deconvoluted into individual, quantifiable steps. This manuscript outlines a comprehensive workflow to identity performance‐limiting steps for a given modification, to facilitate the tailored implementation of mRNA‐based technologies from synthetic biology to therapy.

## 1 BACKGROUND

Synthetic in vitro transcribed messenger RNA (IVT‐mRNA) is increasingly used to overexpress endo‐ or exogenous genes in mammalian cells, both for the purpose of therapeutic use and basic science.^1^, ^2^, ^3^, ^4^, ^5^ The fact that IVT‐mRNA is promptly expressed upon cytoplasmic delivery, without the need for nuclear entry, makes it an appealing alternative to plasmid DNA (pDNA) or virus‐based methods for genetic engineering, especially for non‐dividing primary cells.^6^, ^7^ Moreover, mRNA is mostly considered to be non‐genotoxic. Sporadic reports have recently raised concerns, though, regarding potential reverse transcription and genomic integration of mRNA, exemplified by SARS‐Cov‐2 RNA originating either from vaccination or viral infection. Such integration activity could follow, for example, upon recruitment of the L1 retrotransposon machinery.^8^, ^9^, ^10^ However, such events would be rare and the overall conclusions from the evidence provided are subject to intense debate.^11^, ^12^ In any case, it is safe to argue that—if existing at all—the genotoxic potential of IVT‐mRNA is far lower than that of any kind of viral DNA or RNA vector or that of pDNA. The more traditional concerns about low RNA stability were ameliorated by effective 5′‐end capping, use of optimized 5′ and 3′ untranslated regions (UTRs), and precise control over polyadenylation of 3′ end, all inspired by structural features of natural mRNAs.^1^, ^13^, ^14^, ^15^ Finally, the innate immune response triggered by mRNA when taken up by the prototypical transfection pathway still poses a substantial challenge in mRNA‐based transient genetic engineering. Thus, reliable methods to reconcile robust expression concomitant with absent or tightly controlled immune stimulation are still under scrutiny.^16^, ^17^

Chemical modification of nucleosides is frequently pursued to reduce immunogenicity of IVT‐mRNA.^18^, ^19^ Prominent examples are combined substitution of uridine and cytidine with pseudouridine (Ψ) and 5‐methyl‐cytidine (5meC), which remarkably reduce immunogenicity of the resulting IVT‐mRNA compared to the unmodified variant.^20^ Likewise, we evidenced the same impact of Ψ/5meC modified IVT‐mRNA by transfecting the primary human monocyte‐derived macrophages.^21^ While beneficial for reducing unintended immune responses, low‐level protein production hampered further pursuit of this combined modification in the field. This issue was tackled by using alternative nucleoside modifications, such as 5‐methoxy‐uridine (5moU) and N1‐methyl‐pseudouridine (me^1^Ψ), also in combination with sequence optimization,^22^ and improved manufacturing process.^18^ In an explorative study, we correlated distinct differences of protein production level to transfected IVT‐mRNAs equipped with different 5’ end and nucleoside modifications.^23^ Most notably, Ψ/5meC and 5moU modified IVT‐RNA mediated the lowest and the highest level of protein production, respectively.^23^ The differences in expression levels of modified mRNA can be attributed to distinct ribosomal recruitment and recycling as previously reported.^24^ It was also demonstrated that incorporation of nucleoside modification such as Ψ causes elongation arrest and premature translation termination resulting in production of truncated protein nuclease‐untreated rabbit reticulocyte lysate (RRL) potentially due to shortage of membranous components.^25^ While these studies hint toward possible interference of nucleoside modification with mRNA translation, the impact of other intermediate steps preceding translation remains largely unexplored.^22^, ^26^, ^27^, ^28^, ^29^

**FIGURE 1:**
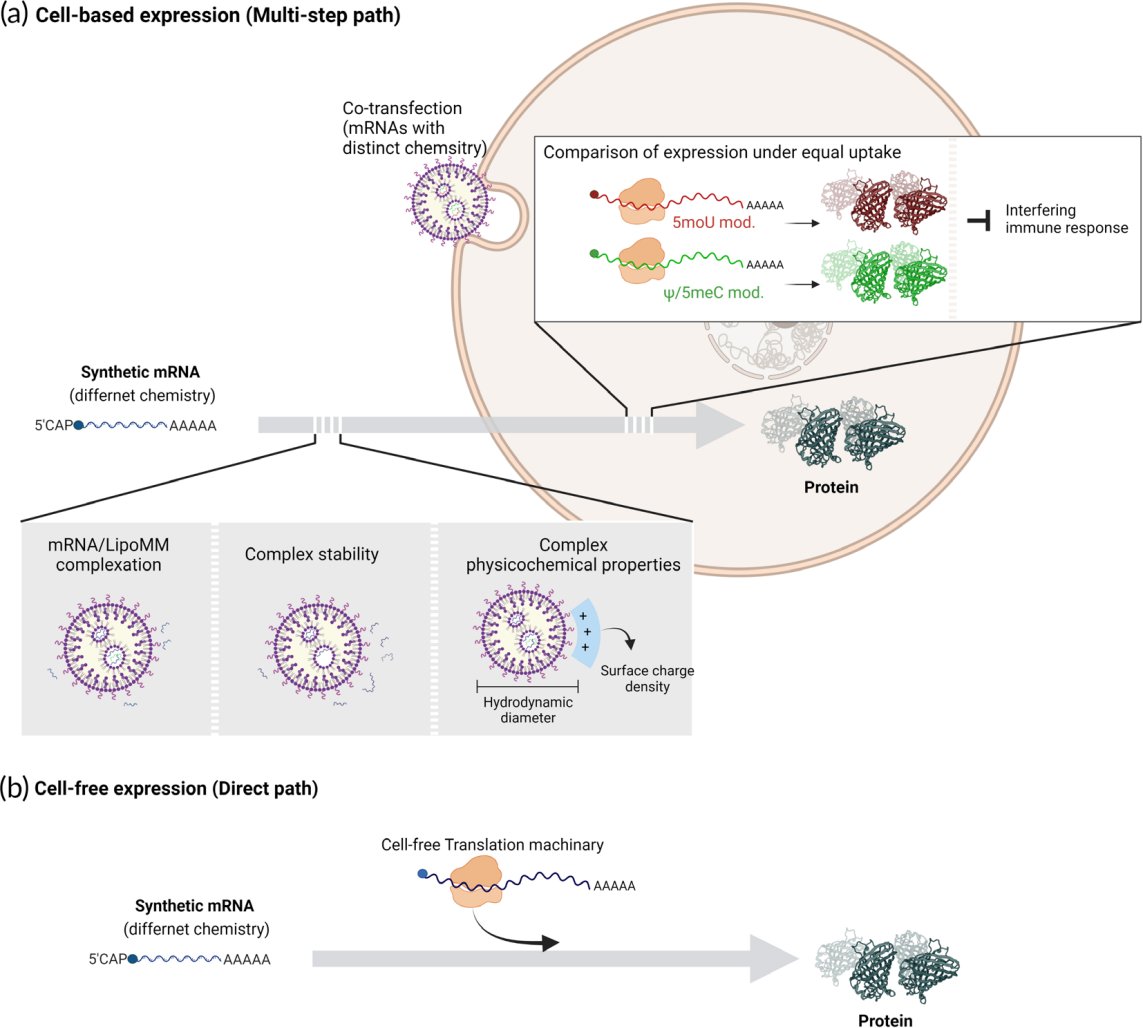
Deconvolution of synthetic mRNA transfection and expression. (a) Distinct steps in cell‐based IVT‐mRNA expression. The gray arrow with partial dashed lines illustrates a multi‐step path, starting with synthetic mRNA as an input material and pointing toward protein as an end‐product. The extracellular steps potentially affected by nucleoside chemistry include complexation of IVT‐mRNA, complex extracellular stability and physicochemical properties, all evaluated in this study. The cellular expression of the two chemically modified IVT‐mRNA, that is 5moU and Ψ/5meC, was compared under equal uptake, enabled by a defined co‐transfection experiment. (b) A cell‐free in vitro translation approach was used to directly evaluate the translational capacity of IVT‐mRNAs with different chemistry, bypassing the extracellular steps as of (a) as well as potential interference by an innate immune response. IVT‐mRNA, in vitro transcribed messenger RNA.

In this study, we aim to establish a workflow to identify individual step(s), which might affect transfection process and eventually result in differences in expression levels depending on IVT‐mRNA chemistry. By introducing quantifiable steps such as complex formation, extracellular stability and physicochemical properties, as well as a cell‐free in vitro assay, we demonstrated that the ribosomal translation is strongly dependent by the employed nucleoside modification, while the nature of the chemical modification does not affect the different formulation steps in our defined experimental set‐up (Figure 1). Both cell‐based (Figure 1a) and in vitro studies (Figure 1b) supported the decisive role of modification‐dependent translatability of IVT‐mRNA.

## 2 RESULTS AND DISCUSSION

By deconvolution of multi‐step cell‐based expression, we examined whether selected nucleoside modifications altered IVT‐mRNA formulation efficiency, extracellular stability, as well as physicochemical properties of the lipoplexes used. Co‐transfection experiments allowed us to correlate transgene expression levels of different chemically modified IVT‐mRNAs with the nucleobase chemistry used, here Ψ/5meC and 5moU, and correlated it to the chemistry of the transcripts, provided that the same amount of each IVT‐mRNA was taken up by individual cells as previously evidenced by us^30^ (Figure 1a). Second, by bypassing the uptake‐related steps using a cell‐free in vitro translation approach, we elucidated the direct impact of nucleoside modification on translational efficacy of IVT‐mRNA (Figure 1b).

The first step for in vitro IVT‐mRNA delivery is its complexation with a carrier, which supports and facilitates its cellular uptake. If the ability of a given carrier to complex IVT‐mRNAs depends on the chemical nature of its nucleosides, it would result in different encapsulation efficiencies, providing cells with different amounts of transcripts to be used as template for protein synthesis.

**FIGURE 2:**
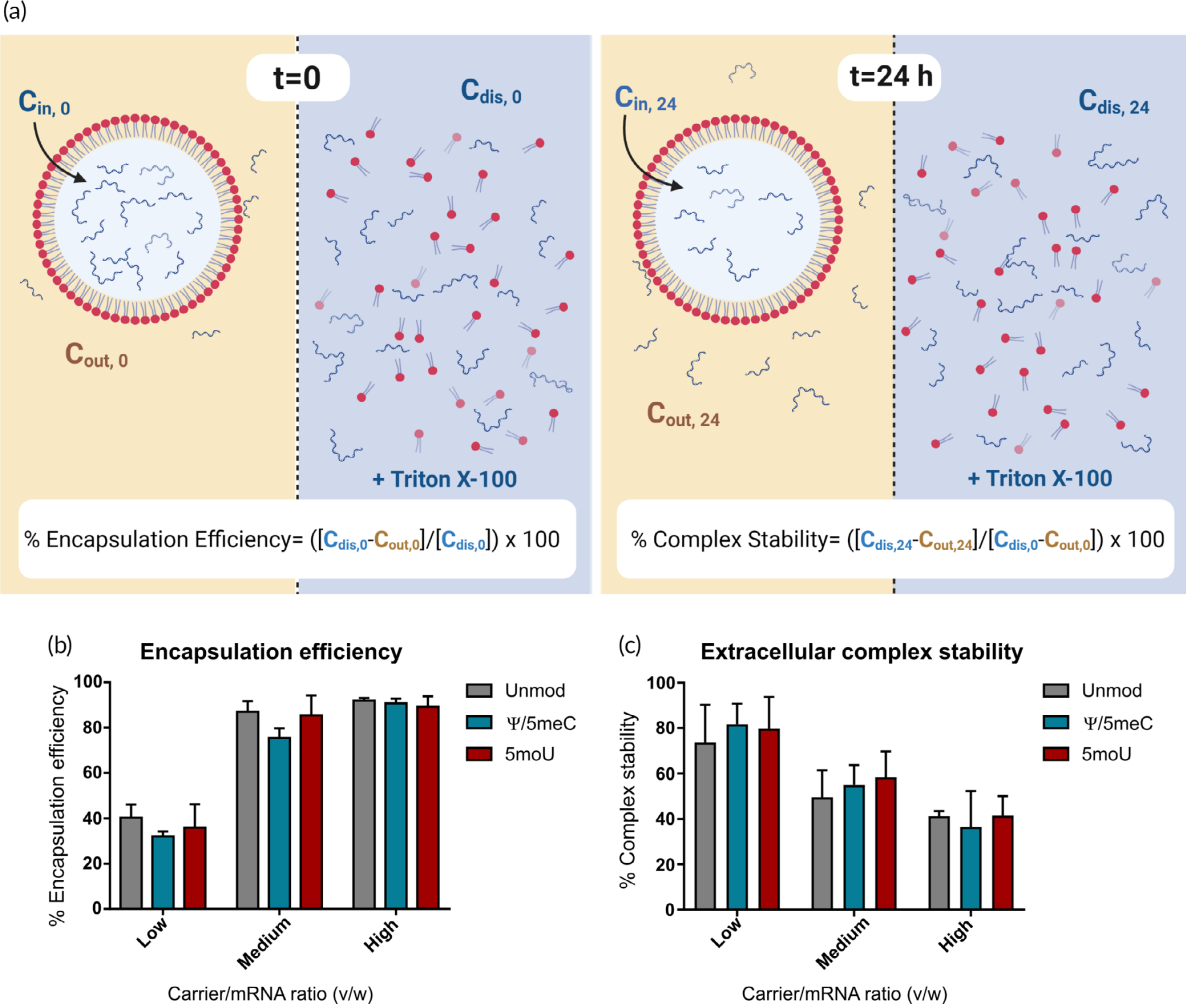
Encapsulation efficiency and extracellular complex stability for IVT‐mRNA with different chemistries. (a) Schematic representation of experiments performed for measurement of encapsulation efficiency (left panel) and extracellular complex stability after 24 h (right panel). Indicated in the white box is the formulas, which were used for calculations. Complexes were dissolved in 5% TritonX‐100 to quantify the total content of IVT‐mRNA. (b) Encapsulation efficiency of different eGFP encoding IVT‐mRNA complexed with liposomal carrier, including unmodified as well as Ψ/5meC and 5moU modified IVT‐mRNA measured by RiboGreen assay. Three different carrier/IVT‐mRNA ratios (v/w) were examined. (c) Extracellular complex stability was measured upon incubation at 37°C in medium for 24 h. The IVT‐mRNA amounts in both complexed and Triton X‐100 treated conditions were quantified by RiboGreen assay. Percent of complex stability was defined as ratio of IVT‐mRNA entrapped within complexes at *t* = 24 h to amount of IVT‐mRNA encapsulated at *t* = 0.

In side‐by‐side experiments, unmodified, and chemically modified Ψ/5meC, and 5moU IVT‐mRNA lipoplexes were formed. Three different lipid‐to‐IVT‐mRNA ratios (v/w) were examined, “medium” referring to the standard transfection conditions routinely used by us, as well as half and twice the amount of IVT‐mRNA input, referred to as “low” and “high,” respectively (Figure 2a, left panel). While the encapsulation efficiency of Ψ/5meC modified IVT‐mRNA was slightly lower than the unmodified and 5moU‐modified IVT‐mRNA, the differences were not substantial (Figure 2b).

As we initially hypothesized, the distinct nucleoside chemistry could potentially impact the strength of IVT‐mRNA‐carrier interactions and thus the extracellular stability of complexes. This notion was empirically examined by incubation of complexes at 37°C for 24 h, substantially longer than the realistic extracellular persistence of lipoplexes in an experimental setting, either in the test tube or in the cell culture medium. Free IVT‐mRNA quantification via RiboGreen assay was employed as described above. The percent of IVT‐mRNA remained within lipoplexes after 24 h respective to IVT‐mRNA initially encapsulated within complexes was defined as extracellular complex stability (Figure 2a, right panel). Implementing this assay and the corresponding formula enabled us to exclude IVT‐mRNA molecules adsorbed on the surface of the complexes, when calculating the amount of IVT‐mRNA protected within liposomal carrier. We identified no differences between the extracellular stability of carriers complexed with distinct IVT‐mRNA chemistries (Figure 2c). However, comparing individual carrier‐to‐IVT‐mRNA ratios, we observed lower complex stability for higher ratios. The underlying reason is unclear to us, but we speculate that complexes with higher initial IVT‐mRNA load are more prone to decomplexation over time. Similarly, the complexes seem to be more stable, when less IVT‐mRNA is packed within lipoplex structure, irrespective of the nucleoside chemistry (Figure 2c).

Overall, neither encapsulation efficiency nor complex extracellular stability was influenced by IVT‐mRNA chemistry, ruling out a prominent contribution to the reported nucleoside modification differences in protein production levels under the experimental conditions chosen by us.

As a next step, we delivered equal amounts of two discernible IVT‐mRNA to cells and to quantify the resulting transgene expression depending on the mRNA chemistry used. To this end, we relied on our previous observation of highly efficient co‐delivery of nucleic acids (NA) via integrated co‐transfection (iCo‐TF) (i.e., premixing of different NA entities before complexation), which was experimentally validated by quantifying downstream protein production via flow cytometry and fluorescent microscopy.^30^ Here, we identified similar encapsulation efficiencies for distinct IVT‐mRNAs chemistries (Figure 2b). Taken together, while the uptake was not directly measured in context of intracellular trafficking mechanism, we concluded that equal amounts of IVT‐mRNA molecules of the same size can be delivered to individual cells by employing the iCo‐TF method, independent of their chemistry or coding sequence.^30^

**FIGURE 3:**
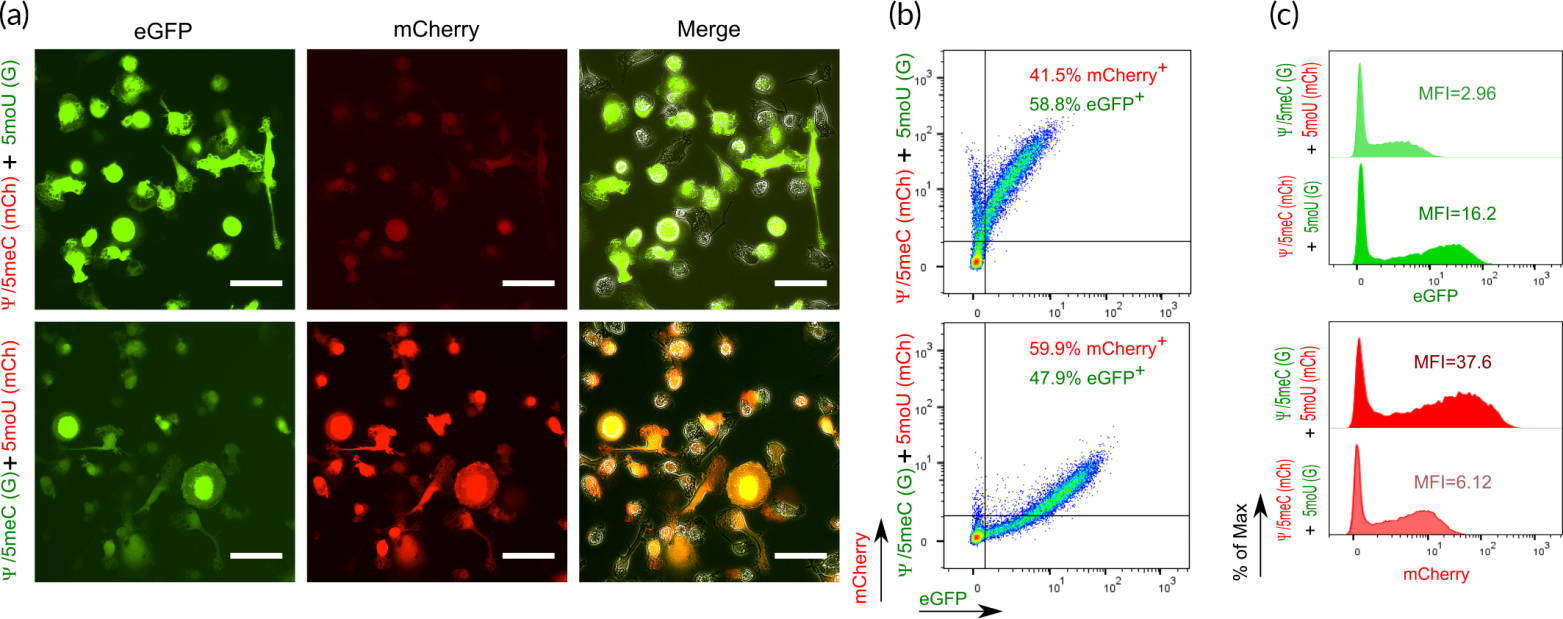
Expression of IVT‐mRNAs modified with either 5moU or Ψ/5meC in a co‐transfection experiment. (a) Representative fluorescent images of macrophages co‐transfected with Ψ/5meC‐modified IVT‐mRNA coding for mCherry and 5moU‐modified IVT‐mRNA coding for eGFP (upper panel), and vice versa (i.e., eGFP IVT‐mRNA modified with Ψ/5meC and mCherry IVT‐mRNA comprising 5moU nucleoside modification) (lower panel), depicted in the eGFP channel, mCherry channel and merge of the two with phase contrast image from left to right (bar = 50 μm). (b) Density plot of the respective co‐transfections; 34.4% and 30.8% of cells remained untransfected in upper and lower dot plots, respectively (not indicated on the graph). (c) Histogram of fluorescent signal intensity of the two markers measured by flow cytometry 24 h after transfection. Data shown are representative for three independent experiments.

Accordingly, we designed co‐transfection experiment by mixing equal amounts of eGFP and mCherry IVT‐mRNA, modified with 5moU and Ψ/5meC, respectively—or vice versa in a reversed modification setup. We chose this strategy because the experimental readout—fluorescent intensity of the reporter proteins cannot be directly compared in parallel given the differences in quantum yield, intracellular half‐life or detection channel sensitivity. In either of the co‐transfection experiments, the transcript modified by 5moU mediated a higher reporter protein signal when compared to the same reporter protein translated from the Ψ/5meC‐modified transcripts in the reciprocal experiment. The experimental outcome, indicating a superior translation capacity for 5moU IVT‐mRNA, was found for both microscopic (Figure 3a) and flow cytometric analysis (Figure 3b). These findings are in line with the previous study by Vaidyanathan et al. who also found 5moU modification outperforming other chemically modified IVT‐mRNAs in terms of level of protein production, which were functionally active, that is, Cas9 protein required for genome editing, measured by formation of indel as readout.^22^

To investigate whether nucleoside chemistry or co‐formulation of the two distinct IVT‐mRNAs influences the physicochemical properties of complexes, size and surface charge were measured. We identified no significant differences between size and zeta potential of lipoplexes containing unmodified, Ψ/5meC modified and 5moU modified mRNA (Figure S1). Likewise, the physicochemical properties of co‐formulated complexes was similar to those with single IVT‐mRNA type, independent of modification scheme (Figure S1).

To rule out the attribution of differences in IVT‐mRNA expression level to potential immune response of cells co‐transfected with the given modifications, the inflammatory cytokine secretion was quantified using multiplex immunoassay. In line with our previous observations,^23^ here we also expected patterns of moderate to negligible immune response for macrophages co‐transfected with 5moU and Ψ/5meC‐modified IVT‐mRNA. No differences were detected between proinflammatory (Figure S2a,c) and antiviral (Figure S2b) cytokines secreted from cells 6 h and 24 h upon co‐transfection with 5moU‐ and Ψ/5meC‐modified IVT‐mRNAs compared to untransfected control, independent of the coding sequence. In contrast, cells transfected with unmodified IVT‐mRNA produced about three orders of magnitude higher proinflammatory as well as antiviral cytokines compared to untransfected cells at both time points. Thus, the quantitative measurement of immune response validated that the observed differences in expression of the IVT‐mRNAs with selected modification did not arise from unintended activation of the transfected macrophages.

**FIGURE 4:**
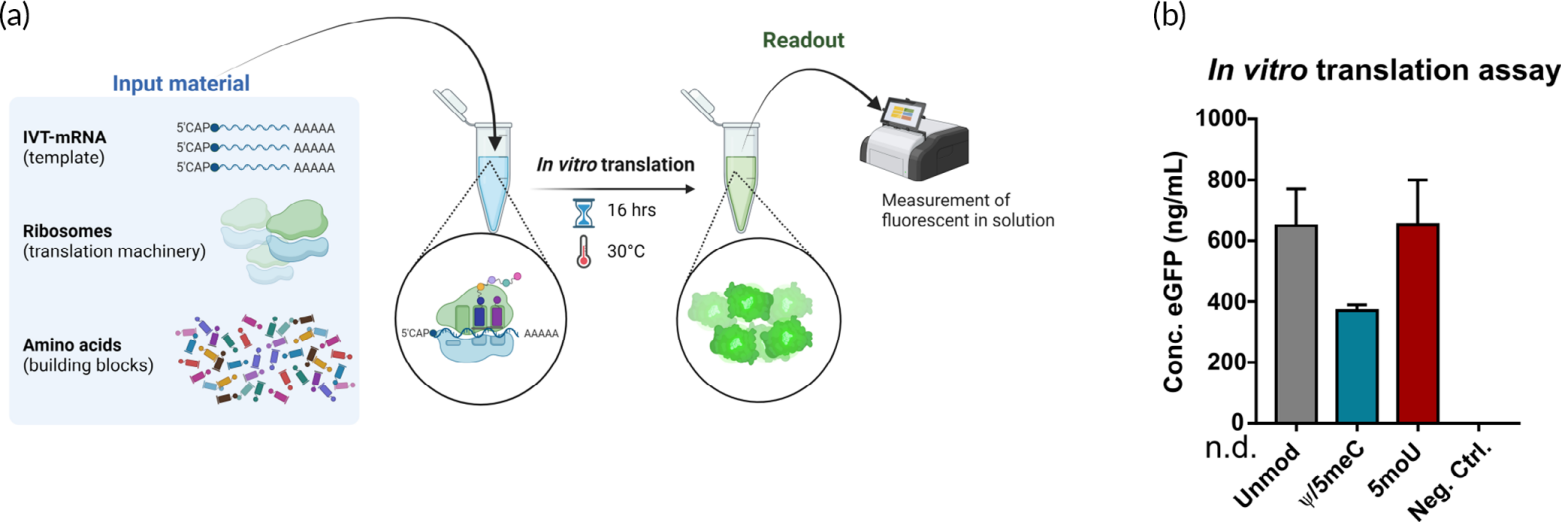
Comparison of translational efficacy of IVT‐mRNA with distinct chemistries. (a) Schematic illustration of in vitro translation assay using capped mRNA as template. (b) Concentration of eGFP protein of cell‐free in vitro translation experiment was measured by fluorescent plate reader and quantified using standard series of eGFP protein with known concentrations. A reaction without template IVT‐mRNA was considered as negative control, where eGFP concentration was non‐detectable (n.d.).

Finally, to be able to directly correlate the expression rate with translational efficacy of chemically modified IVT‐mRNA, we used a cell‐free in vitro translation system. In order to use a commercial human cell‐derived in vitro assay, we had to adapt the original protocol for cap‐dependent translation to find the optimum incubation time and temperature detailed in method part. We examined unmodified, 5moU‐ and Ψ/5meC‐modified IVT‐mRNA coding for eGFP in a side‐by‐side experiment (Figure 4a). The reaction without addition of IVT‐mRNA template served as a negative control. Resulting eGFP protein production levels were measured by fluorimeter and quantified by interpolation from standard curve of serially diluted solutions of recombinant eGFP with known concentrations (Figure 4b). The unmodified and 5moU‐modified IVT‐mRNAs resulted in about two‐fold higher protein production, when compared to Ψ/5meC‐modified IVT‐mRNA (Figure 4b).

This cell‐free assay enabled us to directly correlate the translation efficacy to particular nucleoside chemistries. Implementing a similar in vitro translation method, but using different modification scheme, Svitkin et al.^24^ found a higher translational efficacy for N1‐methyl‐pseudouridine (N1mΨ) modified IVT‐mRNA compared to unmodified IVT‐mRNA. They looked deeper into interaction of translational machinery with transcripts with distinct uridine chemistries. Their findings suggested that increased initiation of translation events mediated by eukaryotic initiation factor 2 (eIF2α), as well as higher ribosome loading density on N1mΨ‐modified IVT‐mRNA compared to other modifications was the underlying mechanism enhancing protein synthesis.^24^

In a more recent study, Svitkin et al. reported that Ψ‐modified mRNA caused ribosome stalling followed by premature translation termination and formation of truncated protein tested in RRL.^25^ The translation elongation arrest, demonstrated in both nuclease‐untreated and more strikingly more pronounced in nuclease‐treated RRL, could be reversed by addition of microsomal membranes and resulted in higher production of full‐size protein.^25^ However, implementing human‐based translation machinery in transfected HeLa cells, we did not encounter the ribosome stalling issue for unmodified compared to modified mRNA. This notion was validated by observing no truncated protein production, when analyzing the integrity of eGFP protein synthesized by either of chemically modified IVT‐mRNAs, as evaluated by immunoblotting using anti‐eGFP antibody (Figure S3). Blotting of the equal amounts of total protein extracted from HeLa cells transfected with eGFP IVT‐mRNA also confirmed the integrity of eGFP signal was not influenced by IVT‐mRNA chemistry (Figures S3 and S4).

Nucleoside chemistry can affect individual steps of IVT‐mRNA delivery, which we addressed in this study for lipoplexes with a given, commercially available lipid component. Any variations in complex physicochemical properties, stability, and uptake and translation efficacy depending on carrier or IVT‐mRNA chemistry can determine protein production levels, typically taken as an endpoint readout. While our results indicate nucleotide chemistry as being key to translatability, especially in in vivo settings where different cell types and tissue effectively compete for uptake of mRNA loaded nanoparticles, the chemistry of the carrier further adds to the complexity. This was addressed in a study by Whitehead and colleagues, who investigated biodistribution of four different lipid formulations complex with five different chemically modified IVT‐mRNAs upon intravenous injection in mice.^31^ They reported distinct patterns of IVT‐mRNA expression in different organs depending on chemical modification of IVT‐mRNA. However, the extent to which these variations were observed mainly altered by carrier chemistry. Most prominently, the detected high protein production rate in monocytic cell lineage in spleen for N1mΨ IVT‐mRNA compared with other chemical modifications including Ψ/5meC, throughout all lipid formulations.^31^ The unique patterns of in vivo biodistribution reported by them might also depend on encapsulation efficiency and stability of complexes, as delineated here in our study. This highlights the importance to go beyond endpoint analysis of protein production and to dig deeper into often neglected, potentially limiting steps in IVT‐mRNA delivery prior to IVT‐mRNA translation.

## 3 CONCLUSION

Our findings provide detailed insights in what causes the strong differences in nucleoside chemistry‐dependent expression of synthetic mRNA, as previously observed by others and us.^22^, ^23^ We highlight the fact that the actual translation, the last step in transgene expression, is only one of multiple steps along the transfection process, where the use of different nucleoside modifications might result in different overall performance. Using a given liposomal carrier, we ruled out that extracellular mRNA complexation and lipoplex stability have a decisive influence. Rather, our experiments in both cellular (transfection) and acellular (in vitro translation) settings point toward nucleoside dependent translatability of synthetic mRNA as the main contributor to modification‐dependent influence on the levels of the encoded protein. Thus, while quantitative or qualitative differences in cellular uptake, endosomal escape, dissociation from carrier, intracellular stability of IVT‐mRNA may also be influenced by mRNA chemistry to some extent, our results support the notion that, at least for the nucleoside modifications analyzed here, differences in ribosomal translation efficiency due the chemical identity of the mRNA template are key to the observed effects. The work presented here outlines an experimental path to facilitate the rational selection of nucleoside modifications in future research from synthetic biology to translational applications.

### 3.1 Methods

Synthetic mRNA was transcribed in vitro from pRNA2‐(A)_128_‐derived pDNA templates, comprising a T7 promoter, a Kozak sequence in the 5’‐UTR followed by the open reading frame of the gene of interest, a head‐to‐tail duplicated human β‐globin 3′‐UTR, and a 128‐base‐pair polyadenine (poly[A]) sequence. Incorporation of the poly(A) sequence in the pDNA template eliminated the requirement for post‐transcriptional 3′‐end adenylation step. Upon linearization and purification of pDNA template, as previously described,^30^ mRNA was synthesized by TranscriptAid T7 High Yield Transcription Kit (Thermo Fisher Scientific, Germany) according to the manufacturer's instruction. The 5′‐end of IVT‐mRNA constitutes an anti‐reverse cap analog (ARCA; Jena Bioscience, Germany). The 5moU IVT‐mRNA was synthesized with complete substitution of uridine with 5‐methoxy‐uridine (Jena Bioscience), and for Ψ/5meC IVT‐mRNA uridine and cytidine were entirely substituted with pseudouridine and 5‐methyl‐cytidine (Jena Bioscience), respectively. The transcripts were purified by precipitation using lithium chloride, and subsequently resuspended in 0.1 mM EDTA solution in UltraPure™ nuclease‐free sterile water (Merck Millipore, Germany). The integrity of IVT‐mRNA products was evaluated by denaturing agarose gel electrophoresis, and the concentration was measured by UV/Vis‐spectroscopy (NanoDrop 1000 Spectrophotometer; Peqlab, Germany).

IVT‐mRNA was complexed with Lipofectamine MessengerMAX (LipoMM; Thermo Fisher Scientific) as previously described.^21^ Briefly, the MessengerMAX reagent was diluted 1:50 (vol) in Opti‐MEM reduced serum medium (Thermo Fisher Scientific). Upon 10‐min incubation at RT, the LipoMM solution was added to the equal volume of Opti‐MEM containing 4 ng·μL^−1^ IVT‐mRNA. The mixture was shortly vortexed and incubated for 5 min at RT.

The encapsulation efficiency of IVT‐mRNA within LipoMM complexes were measured quantitatively by Quant‐iT RiboGreen assay kit (Thermo Fisher Scientific), according to previously published protocol.^30^ Nucleic acid/lipid complexes with different IVT‐mRNA/LipoMM (w/v) ratios were prepared by mixing 1:100, 1:50, and 1:25 dilutions of LipoMM, with constant amounts of IVT‐mRNA, here referred to as low, medium, and high, respectively. The complexes were subsequently diluted in 1× TE assay buffer (10 mM Tris–HCl, 1 mM EDTA, pH 7.5) to final volume of 100 μL, mixed with equal volume of RiboGreen dye 200‐fold diluted in 1× TE buffer. Parallel samples were prepared and then dissociated with 5 μL of 10% Triton X‐100 (Figure 2a, left panel). All samples were incubated for 20 min at 37°C in the dark. TE buffer only was used as blank. Fluorescence was measured using a Tecan Infinite 200 PRO plate reader at 485/535 nm (Ex/Em).

The influence of nucleoside modification on encapsulation efficiency of IVT‐mRNA was quantified by employing the RiboGreen fluorescence assay, capable to discriminate between accessible (free or surface‐bound IVT‐mRNA) and non‐accessible, complexed IVT‐mRNA.

The encapsulation efficiency was calculated using the following formula:

$$\%\;\text{Encapsulation efficiency}=\left(\left[C_{\mathrm{dis},0}-C_{\text{out},0}\right]/\left[C_{\mathrm{dis},0}\right]\right)\times 100$$

where C_dis,0_ is the concentration of IVT‐mRNA in complexes dissolved in Triton‐X100 and C_out,0_ is the concentration of free IVT‐mRNA in intact complexes measure at *t* = 0, that is immediately after complex formation (Figure 2a, left panel).

The extracellular stability of lipoplexes were calculated according to the following formula:

$$\%\;\text{Extracellular complex stability}=\left(C_{\text{inside},24}/C_{\text{inside},0}\right)\times \;100$$

where C_inside,t_ is defined as:

$$C_{\text{inside},t}=C_{\mathrm{dis},t}-C_{\text{out},t}$$

where C_dis,t_ is the concentration of IVT‐mRNA in complexes dissolved in Triton‐X100 and C_out,t_ is the concentration of free IVT‐mRNA in intact complexes measure at time = t h (Figure 2a, right panel).

Dynamic light scattering was employed to measure the particle size and zeta potential of complexes via a Zetasizer Nano (Malvern Instruments, Herrenberg, Germany).

Co‐transfection was performed with complexes prepared with mixture of eGFP and mCherry coding IVT‐mRNAs with different modifications, that is 5moU‐mRNA coding for eGFP with Ψ/5meC IVT‐mRNA coding for mCherry, and vice versa. Primary human macrophages were derived from monocytes isolated from peripheral blood mononuclear cells according to our previously published protocol.^21^ Buffy coats obtained from Deutsche Rote Kreuz, Berlin; ethics vote EA1/364/21; Charité University Medicine Berlin. Macrophages were co‐transfected with complexes containing 250 ng total IVT‐mRNA, which was added to each well of six‐well plate with cell density of 2.00E+06 cells, in very low endotoxin RPMI medium (PAN Biotech) supplemented with 10 vol% FBS. Cells were evaluated 24 h post‐transfection both with fluorescent microscopy imaging and flow cytometry, for qualitative and quantitative assessment of protein synthesis, respectively. In this regard, cells were imaged with an ELIPSE Ti‐U inverted microscope (Nikon) equipped with a mercury light source, Intensilight C‐HGFI, and single‐band filter sets; eGFP‐BP filter (466/40, 525/50 nm), or TRITC filter (562/40, 641/75 nm) (Semrock, Germany). All images were analyzed with NIS‐Elements imaging software, version 4.51 (Nikon). Flow cytometric measurements were performed upon harvesting cells by scraping, via a MACSQuant VYB® flow cytometer (Miltenyi Biotec, Germany). All flow cytometric data were analyzed with FlowJo software V10.

The cytokine secretion was analyzed using Bio‐Plex immunoassays (Bio‐rad, Germany), using Bio‐Plex standards including Pro Human Cytokine Screening Group 1 (171D50001; Bio‐rad) for TNF‐α and IL‐6, and Pro Human Inflammation Panel 1 (171DL0001) (Bio‐rad) for IFN‐β, according to the respective manufacturers protocol. Briefly, macrophages culture media were collected at 6 h and 24 h post transfection was added to diluted (1×) magnetic beads conjugated with capture antibody in a 96‐well assay plate, in parallel with dilution series of standards, and incubated on a shaker at 900 rpm at RT for either 30 min or 1 h, depending on the assay type. Subsequently, samples were incubated with 1× biotinylated detection antibodies, followed by 1× PE‐conjugated streptavidin, and incubated on a shaker at 900 rpm at RT for 30 min, and 10 min, respectively. Three times washing was performed between each step. After the last washing steps, beads were resuspended in 125 μL assay buffer, shaken at 900 rpm for 30 s, and cytokines quantified (Bio‐Plex 200 System; Bio‐rad).

The cell‐free in vitro translation was performed using 1‐Step Human Coupled IVT kit (Thermo Fisher Scientific), which harnesses HeLa cell extract to enable full length protein synthesis from IVT‐mRNA as well as pDNA templates. Being optimized for cap‐independent internal ribosomal entry site‐comprising RNA templates, the manufacturers' protocol was modified to accommodate translation of ARCA capped IVT‐mRNA, according the recommendations provided by Thermo Fischer Scientific. IVT‐mRNA templates for translation were heat denatured for 5 min at 60°C, and subsequently incubated on ice for 5 min. The reaction mixture was prepared according to manufacturer's instruction using 2.5 μg IVT‐mRNA per reaction, and incubated at 30°C for 16 h. The pDNA, pCFE‐GFP, supplemented with kit was used as positive control and a negative control reaction by addition of DEPC‐water rather than template. The eGFP protein synthesis was quantified by fluorescent measurement employing a Tecan Infinite 200 PRO plate reader with an excitation wavelength of 485 nm and emission wavelength of 535 nm. The concentration of eGFP protein was subsequently interpolated from standard curve made by series of dilutions of Pierce™ Recombinant GFP Protein (Thermo Fisher Scientific) with known concentrations.

Data are presented as means ± standard deviation (SD) of at least three independent experiments. In case of primary cells, cells were derived from at least three different donors. Two‐way ANOVA test was performed for multiple comparisons between different groups with a 95% confidence interval. Statistical significance was considered as *p* < 0.05. Data were statistically analyzed via Prism 7.00 software (GraphPad, USA). Illustrations in Figures 1, 2, and 4 were created with BioRender.com.

## AUTHOR CONTRIBUTIONS

**Hanieh Moradian:** Conceptualization (equal); data curation (lead); formal analysis (lead); investigation (lead); methodology (lead); project administration (equal); visualization (lead); writing – original draft (lead). **Marko Schwestka:** Investigation (supporting); methodology (supporting). **Toralf Roch:** Formal analysis (supporting); writing – review and editing (supporting). **Manfred Gossen:** Conceptualization (equal); funding acquisition (lead); resources (lead); supervision (lead); writing – review and editing (lead).

## CONFLICT OF INTEREST STATEMENT

The authors declare no conflicts of interest.

### PEER REVIEW

The peer review history for this article is available at https://www.webofscience.com/api/gateway/wos/peer-review/10.1002/btm2.10622.

## Supporting information


**DATA S1.** Supporting Information.

## Data Availability

The datasets generated during and/or analysed during the current study are available from the corresponding author on reasonable request.
