# Supplementary material for: Deconvolution of synthetic mRNA expression: Nucleoside chemistry alters translatability
Source: Bioeng Transl Med. 2023 Nov 28;9(4):e10622. doi: 10.1002/btm2.10622 (PMC11256140; doi:10.1002/btm2.10622)
Supplement: Supplementary file 1 — DATA S1. Supporting Information. [file BTM2-9-e10622-s001.docx]

**Deconvolution of synthetic mRNA expression: nucleoside chemistry alters translatability**

Hanieh Moradian^1, 2^, Marko Schwestka^1,2^, Toralf Roch ^2,3^ Manfred Gossen^1, 2, ^[[1]](#footnote-1)^^

1. Institute of Active Polymers, Helmholtz-Zentrum Hereon, 14513 Teltow, Germany
2. Berlin Institute of Health Center for Regenerative Therapies (BCRT), 13353, Berlin, Germany
3. CheckImmune GmbH, Campus Virchow Klinikum, Cranach Haus, 13353 Berlin, Germany

* Corresponding Author, email address: manfred.gossen@hereon.de

**Fig. S1. Physicochemical properties of lipoplexes formulated with different IVT-mRNA chemistries**

Particle size and zeta potential of lipoplexes measure by dynamic light scattering (DLS) indicated on left and right y-axis, respectively. Lipoplexes were made with IVT-mRNA coding for eGFP encompassing unmodified nucleotides (G nm), 5-methoxy-uridine-modified nucleosides (G 5moU) and combined modification with pseudouridine and 5-methyl-cytidine (G Ψ/5meC). Side-by-side are complexes co-formulated with mCherry coding IVT-mRNA modified with 5moU and eGFP coding IVT-mRNA modified with Ψ/5meC (mCh 5moU+ G Ψ/5meC) and mixture of mCherry coding IVT-mRNA modified with Ψ+5meC and eGFP coding IVT-mRNA modified with 5moU (mCh Ψ/5meC + G 5moU).


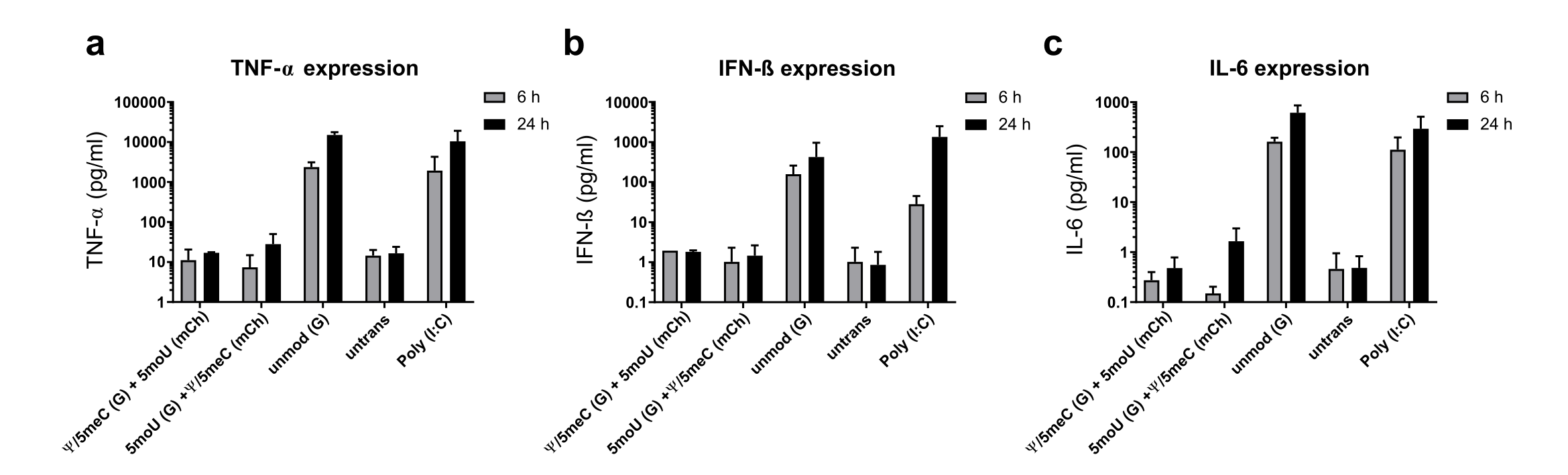


**Fig. S2.** **Cytokine secretion of co-transfected cells**

**(a)** TNF-α, **(b)** IFN-β, and **(c)** IL-6 secretion measured 6 h and 24 h after transfection. Poly (I:C) was implemented as positive control. Unmodified mRNA coding eGFP is indicated for comparison. Values are presented as mean ± SD, n = 3. Error bars indicate SD.


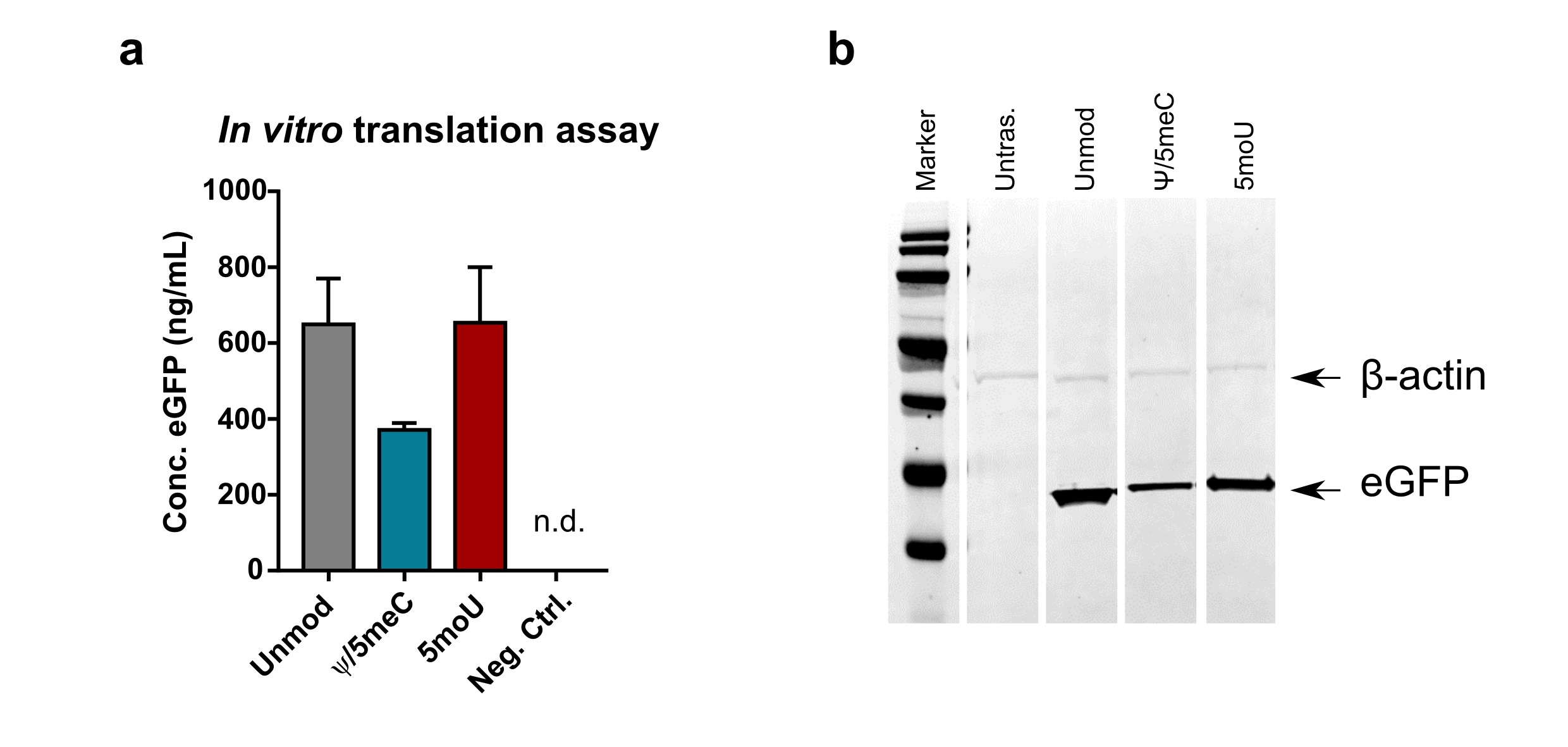


**Fig. S3.** **Evaluation of IVT-mRNA coded eGFP integrity and relative production level by western blot**

Western blot of HeLa cells transfected with unmodified, as well as Ψ/5meC and 5moU modified IVT-mRNA. β-actin was used as a housekeeping control. The uncropped image of the membrane is presented in ***Fig. S4***. (Unmod: unmodified, Ψ: pseudouridine, 5meC: 5-methyl-cytidine, and 5moU: 5-methoxy-uridine).


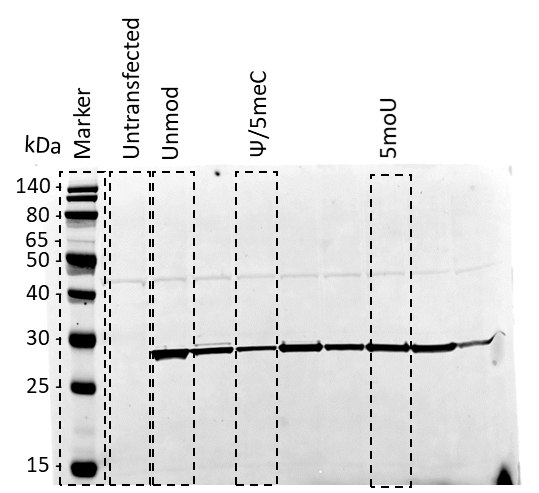


**Fig. S4.** **Uncropped image of western blot membrane of the samples presented in Fig. S4**

Immunoblot lanes depicted in Fig. S4 are highlighted by dashed boxes. The eGFP protein (26 kDa) and the housekeeping protein β-actin (44 kDa) migrated at the expected position. Equal amounts of total protein were loaded per lane.

1. Corresponding author: Dr. Manfred Gossen, Helmholtz-Zentrum Hereon, Institute of Active Polymers, Kantstr. 55, 14513 Teltow, Germany, and Berlin-Brandenburg Center for Regenerative Therapies, Charité Virchow Campus, Berlin. Tel.: +49 (0) 30 450539-491; Fax: +49 (0)30 450539-991; E-mail: manfred.gossen@hereon.de [↑](#footnote-ref-1)
